# Effect of a self-care program among women with urinary incontinence: A quasi-experimental study

**DOI:** 10.25122/jml-2022-0297

**Published:** 2023-07

**Authors:** Magda Soliman, Mona El-Sheikh, Nadia Farrag

**Affiliations:** 1 Department of Laboratories, Faculty of Nursing, Ain Shams University, Cairo, Egypt; 2 Department of Maternity and Gynecological Nursing, Faculty of Nursing, Ain Shams University, Cairo, Egypt

**Keywords:** program, women, self-care, urinary, incontinence, practice

## Abstract

Urinary incontinence is a multifactorial health problem that significantly affects women's quality of life. This quasi-experimental pre/post-test study aimed to evaluate the effect of a self-care program on women suffering from urinary incontinence. The study was conducted at the urodynamic unit within Ain Shams Maternity University Hospital, with a purposive sample of 100 women diagnosed with urinary incontinence, with no medical or surgical conditions, and who were not pregnant. A structured interview questionnaire and women's self-care practices checklists were used as study tools. The results showed that 72.0% of the study group and 68.0% of the control group were housewives. Significantly improved self-care practices and bladder retraining were observed in the study group compared to the control group post-intervention and during follow-up (p<0.001). Furthermore, the study group demonstrated a reduction in the severity of urinary incontinence and improved health practices, whereas the control group did not exhibit significant changes. These findings emphasize the significance of self-care programs in managing urinary incontinence and enhancing women's quality of life.

## INTRODUCTION

Urinary incontinence (UI) is a prevalent condition that significantly impacts the quality of life of individuals worldwide, particularly women. The incidence of UI is higher in women compared to men, with varying prevalence rates across different populations [1]. UI is classified into stress urinary incontinence, urge urinary incontinence, and mixed urinary incontinence, with severity ranging from minor leakage to uncontrollable wetting [2]. Despite its prevalence, UI is often underreported, making it challenging to obtain accurate statistics [3].

Risk factors for UI in women include pregnancy, vaginal childbirth, pelvic surgery, obesity, and aging [4]. It can have a profound negative impact on various aspects of a woman's life, including daily activities, sexual and interpersonal relationships, and emotional well-being [5]. However, many women do not seek medical attention for UI, considering it a normal part of childbirth and aging rather than a significant health problem [6].

Effective treatment options for UI include surgery, substance rehabilitation, behavioral treatments, and biofeedback. Noninvasive techniques like pelvic muscle and rectal balloon exercises have also been suggested as essential remedies for UI. Behavioral therapy is a preferred method for stress UI, with a similar rate of improvement to pharmacotherapy [7]. Several studies have systematically reviewed self-care practices targeting pelvic floor muscles for UI management [8, 9]. Nurses play a crucial role in promoting urinary continence by utilizing research-based practices, providing education and training, and implementing high-quality care [4].

The Middle East is mainly affected by UI, a stigmatizing disorder that can impair women's quality of life and limit their daily activities [10]. Barriers to seeking care for UI include feelings of embarrassment and shame and misconceptions about the condition being incurable. Addressing these barriers, raising awareness about available treatment options, and providing accurate information and support to encourage women to seek help [11].

Standardized and validated questionnaires can help identify urinary incontinence and initiate discussions and treatments in healthcare settings. By implementing routine screening protocols for urinary incontinence, healthcare providers can increase awareness and education about this condition and improve the quality of care for affected women [8]. Additionally, self-care plays a vital role in maintaining physical, mental, and emotional health, enabling individuals to manage and adapt to various health-related situations [12].

This study aimed to evaluate the effectiveness of a self-care program in improving UI among women. The research hypothesis was that the implementation of a self-care program would lead to a reduction in UI symptoms and an improvement in women's quality of life. By addressing this significant health issue, we can alleviate the financial burden associated with clinical and surgical interventions and enhance the productivity and daily functioning of individuals affected by UI [13, 14]. This study is particularly relevant in the context of Ain Shams Maternity Hospital, where a considerable number of women with UI seek healthcare services [15, 16].

## MATERIAL AND METHODS

### Study design and setting

This study employed a quasi-experimental design to compare the effects of a self-care program on women suffering from urinary incontinence. The research was conducted at the urodynamic unit of Ain Shams Maternity University Hospital, a specialized hospital affiliated with Ain Shams University. The hospital, established in 1928, is located in the Abbasia district and provides cost-free maternal and child health services. With approximately 3200 beds, the hospital serves a large number of patients annually through its outpatient clinics and internal departments.

### Participants

A purposive sample of women suffering from urinary incontinence was recruited for the study. The inclusion criteria required participants having been diagnosed with urinary incontinence, free from medical and surgical problems, educated, and not pregnant. 100 participants were recruited between 2020 and 2021 at the urodynamic unit of Ain Shams Maternity University Hospital. The participants were divided into the study group (n=50) and the control group (n=50). The study group received the self-care program, an intervention guideline booklet in Arabic. This program aimed to enhance self-care care among women diagnosed with stress UI by improving their knowledge, practices, and attitudes regarding urinary incontinence. The control group received medication and routine care without any additional intervention.

#### Intervention

The overarching objective of the intervention was to enhance participants' knowledge, practices, and attitudes. The program was structured into six distinct sessions, each targeting specific goals:

Session (1): The initial session aimed to provide participants with a comprehensive overview of the program and its underlying objectives.
Session (2): This session aimed to equip participants with general insights into the meaning, types, causes, predisposing factors, complications, various diagnostic methods, and therapeutic approaches associated with urinary incontinence.
Session (3): Participants were guided to foster self-control behaviors and apply the program's principles in this session.
Session (4): This session focused on training participants in bladder retraining techniques and the effective utilization of pads.
Session (5): Participants engaged in practicing Kegel exercises, adopting pubic area hygiene measures, and maintaining a fluid chart that tracked input and output.
Session (6): The final session emphasized the accurate and successful application of the guideline program.

### Data collection tools

Three tools were utilized to collect data for the study. The first tool was a structured interview questionnaire developed by the researcher after conducting a comprehensive literature review. The questionnaire consisted of multiple-choice, open-ended, and close-ended questions and was divided into three parts. The first part collected information on the general characteristics of the participants, including age, place of residence, occupation, educational level, and socioeconomic status. The second part focused on participants' obstetric, gynecological, urological, and surgical history. The third part assessed the participants' self-care level regarding urinary incontinence and the high-risk group before and after the intervention program. Two scoring systems were used to rate the total level of self-care and the level of total practicing self-care. A follow-up sheet was also designed by the researcher to track the use of guidelines among women with urinary incontinence. Additionally, a sample frequency volume chart/voiding diary was used to monitor changes in intake, urine passed, and urine leakage before and after using the program [17].

### Statistical analysis

The data collected in this study was subjected to various statistical analyses to explore relationships and patterns among the variables. Descriptive statistics, such as means and standard deviations, were used to summarize continuous variables like age, BMI, and symptom severity, providing a clear overview of the distribution and central tendencies within the study and control groups. Additionally, chi-square tests and ANOVA were employed to assess associations and differences between groups.

## RESULTS

### Sociodemographic characteristics

The mean age of the study group was 28.84 years (SD=5.34), while the mean age of the control group was 30.09 years (SD=5.43) (Table 1). Educational levels varied, with 40.0% of the study group and 56.0% of the control group having completed preparatory education, while 32.0% of the study group and 20.0% of the control group had attained a university or postgraduate education. The majority of both groups were housewives, with 72.0% of the study group and 68.0% of the control group reporting this as their current job status. In terms of marital status, 80.0% of the study group and 72.0% of the control group were married, while 20.0% of the study group and 28.0% of the control group were not married. A significant proportion of both groups were classified as obese based on their body mass index (BMI), with 44.0% of the study group and 52.0% of the control group falling into this category. No statistically significant differences were observed between the two groups across all socio-demographic characteristics.

**Table 1:** Demographic characteristics of study groups (n=100)

Item	Study		Control		Total	
	N=50		N=50		N=100	
	No.	%	No.	%	No.	%
**Age (years)**						
18-27.9	18	36.0	14	28.0	32	32.0
28-37.9	20	40.0	20	40.0	40	40.0
38-47.9	4	16.0	6	12.0	10	10.0
48-60	8	16.0	10	20.0	18	18.0
Mean±SD	28.84±5.34		30.09±5.43		29.46±5.39	
**Educational level**						
Preparatory education	20	40.0	28	56.0	48	48.0
Secondary education	14	28.0	12	24.0	26	26.0
University and postgraduate	16	32.0	10	20.0	26	26.0
**Current job**						
Housewife	36	72.0	34	68.0	70	70.0
Crafts job	6	12.0	6	12.0	12	12.0
Employee	8	16.0	10	20.0	18	18.0
**Marital status**						
Married	40	80.0	36	72.0	76	76.0
Not married	10	20.0	14	28.0	24	24.0
**Body Mass Index**						
Normal weight (18.5–25)	16	32.0	12	24.0	28	28.0
Overweight (25–30)	12	24.0	12	24.0	24	24.0
Obesity ( >30)	22	44.0	26	52.0	48	48.0
Mean±SD	32.14±8.14		34.25±9.17		33.27±8.44	

### Pregnancy and delivery characteristics

Pregnancy and delivery-related information is presented in Table 2. Gravidity data showed that 76.0% of the study group had three pregnancies, while 68.0% of the control group had three or more pregnancies. As for parity, 52.0% of the participants in the study group had delivered twice, and 40.0% of participants in the control group had delivered three times. Most participants in both groups experienced a single abortion (36.0%). Cesarean section was the most common method of birth for the study group (36.0%), while the control group had a higher rate of normal vaginal delivery (40.0%). Instrumental deliveries, such as forceps and ventouse deliveries, were reported by both groups with no significant differences. A majority of participants in both groups experienced problems during previous deliveries (76.0% in the study group and 80.0% in the control group, respectively). Within the study group, 40.0% of the participants reported experiencing incontinence, while in the control group, 30.0% reported excessive bleeding.

**Table 2:** Obstetrics and gynecological history (in both groups n=100)

Obstetrics and gynecological history	Study		Control		Total	
	N=50		N=50		N=100	
	No.	%	No.	%	No.	%
**Gravidity**						
1	8	16.0	4	8.0	12	12.0
2	4	8.0	12	24.0	16	16.0
Three or more	38	76.0	34	68.0	72	72.0
**Parity**						
1	12	24.0	10	20.0	22	22.0
2	26	52.0	20	40.0	46	46.0
Three or more	12	24.0	20	40.0	32	32.0
**Number of abortions**						
1	18	36.0	20	40.0	38	38.0
2	16	32.0	16	32.0	32	32.0
Three or more	16	32.0	14	28.0	30	30.0
**Type of delivery**						
Normal vaginal delivery	16	32.0	20	40.0	36	36.0
Cesarean section (CS)	18	36.0	16	32.0	34	34.0
Forceps delivery	10	20.0	9	18.0	19	19.0
Ventouse delivery	6	12.0	5	10.0	11	11.0
**Problems associated with previous deliveries**						
No	12	24.0	10	20.0	22	22.0
Yes: -Excessive bleeding (hemorrhage) -Infection or sepsis -Incontinence	(38) 6 12 20	76.0 15.8 31.6 52.6	40 15 14 11	80.0 37.5 35.0 27.5	78 21 26 31	78.0 26.9 33.3 39.7

### Symptoms of urinary incontinence

Table 3 provides information on the symptoms of urinary incontinence reported by the study and control groups. The most common symptoms in the study group were urine leaks with effort (96.0%), urine leaks during sexual intercourse (60.0%), feeling embarrassed (76.0%), isolating oneself (40.0%), limiting work and social life (72.0%), going to pee more than eight times a day (52.0%), and getting up more than twice at night to urinate (40.0%). Similarly, in the control group, the most common symptoms were urine leaks with effort (96.0%), urine leaks during sexual intercourse (64.0%), feeling embarrassed (80.0%), isolating oneself (80.0%), limiting work and social life (32.0%), going to pee more than eight times a day (60.0%), and getting up more than twice at night to urinate (40.0%).

**Table 3:** Distribution of urinary incontinence symptoms across study groups (n=100)

Symptoms of urinary incontinence	Study		Control		Total	
	No.	%	No.	%	No.	%
Urine leak with effort	48	96.0	48	96.0	96	96.0
Urine leak during sexual intercourse	30	60.0	32	64.0	62	62.0
Feeling embarrassed and isolating oneself	38	76.0	40	80.0	78	78.0
Limiting work and social life	38	76.0	40	80.0	78	78.0
Urinating more than 8 times a day	20	40.0	16	32.0	36	36.0
Gettin up more than twice a night to urinate	36	72.0	30	60.0	66	66.0

### Voiding diary and self-care

Table 4 presents the results of the voiding diary items and self-care practices. There was a highly statistically significant difference between the study and control groups in all items of the voiding diary during the post-program assessment (p<0.001). Similarly, during the follow-up assessment, there was a statistically significant difference between the study and control groups across all voiding diary items (p<0.05). However, during the pre-program assessment, there was no statistically significant difference between the study and control groups across all voiding diary items (p>0.05).

**Table 4:** Distribution of voiding diary items/day (both groups n=100)

Voiding diary/day 750 ml-1375ml (3-5.5 cups) 1500ml-2500ml 6-10			Pre (n=50)			Post (n=50)			Follow up			Chi-square test					
			>2500ml >10 cups	750 ml-1375ml (3-5.5 cups)	1500ml-2500ml 6-10	>2500ml >10 cups	750 ml-1375ml (3-5.5 cups)	1500ml-2500ml 6-10	>2500ml >10 cups	Pre		Post		Follow Up			
										x^2^	p	x^2^	p	x^2^	p		
Fluids intake, the exact amount in ml	Study (n=50)	No.	4	20	26	32	12	6	30	11	9	0.919	0.632	23.236	<0.001**	8.611	0.021*
		%	8.0	40.0	52.0	64.0	24.0	12.0	60.0	22.0	18.0						
	Control (n=50)	No.	6	16	28	9	20	21	7	22	21						
		%	12.0	32.0	56.0	18.0	40.0	42.0	14.0	44.0	42.0						
Amount of urine excreted, the exact amount in ml	Study (n=50)	No.	2	28	20	24	16	10	22	16	12	1.070	0.586	15.569	<0.001**	9.370	0.009*
		%	4.0	56.0	40.0	48.0	32.0	20.0	44	32	24						
	Control (n=50)	No.	4	24	22	6	25	19	8	23	19						
		%	8.0	48.0	44.0	12.0	50.0	38.0	16.0	46.0	38.0						
Amount of stress urinary incontinence, the exact amount in ml	Study (n=50)	No.	7	19	24	26	18	6	20	21	9	0.385	0.833	18.778	<0.001**	9.016	0.011*
		%	14.0	38.0	48.0	52.0	36.0	12.0	40	42	18						
	Control (n=50)	No.	6	17	27	8	20	22	10	18	22						
		%	12.0	34.0	54.0	16.0	40.0	44.0	20.0	36.0	44.0						
			**1-3 times**	**3-6 times**	**>6 times**	**1-3** **times**	**3-6 times**	**> 6 times**	**1-3 times**	**3-6 times**	**> 6 times**						
Frequency of leak	Study (n=50)	No.	3	27	20	26	17	7	23	17	10	1.22	0.545	17.673	<0.001**	9.879	0.007*
		%	6	54	40	52	34	14	46	34	20						
	Control (n=50)	No.	5	22	23	7	24	19	9	21	20						
		%	10	44	46	14	48	38	18	42	40						
Total	Study (n=50)	No.	4	24	22	27	16	7	24	16	10	0.666	0.717	17.521	<0.001**	10.827	0.005*
		%	8.0	48.0	44.0	54.0	32.0	14.0	48.0	32.0	20.0						
	Control (n=50)	No.	5	20	25	8	22	20	9	21	20						
		%	10.0	40.0	50.0	16.0	44.0	40.0	18.0	42.0	40.0						

p-value >0.05 is insignificant; *p-value <0.05 is significant; **p-value <0.001 is highly significant.

- pre (before implementing the program)

- post (after implementing the program)

- follow-up (3 months after implementing the program)

Table 5 provides information on the statistical significance between the study group and control group in various self-care items. There was a high statistical significance between the study group and control group in all items of bladder retraining. Furthermore, there was a highly statistically significant difference between the study group and the control group in pelvic floor muscle exercise after implementing the intervention and during follow-up assessments (p<0.001). Similarly, there was a statistically significant difference between the study and control groups in hygienic measures during the post-program and follow-up assessments (p<0.001). However, there was no statistically significant association between the study group and the control group in practicing hygienic measures before the intervention (p>0.05).

**Table 5:** Distribution of the study of women’s self-care concerning their Domains of self-care (in both groups n=100)

Domains of self-care		Study Group				Control Group				Chi-square test	
	Satisfactory		Unsatisfactory		Satisfactory		Unsatisfactory				
	No.	%	No.	%	No.	%	No.	%	x2	p-value	
Bladder retraining	Pre	10	20.0	40	80.0	8	16.0	42	84.0	2.687	0.319
	Post	45	90.0	5	10.0	17	34.0	33	66.0	21.602	<0.001**
	Follow up	45	90.0	5	10.0	15	30.0	35	70.0	18.583	<0.001**
Pelvic floor muscle exercise	Pre	12	24.0	38	76.0	10	20.0	40	80.0	2.771	0.312
	Post	42	84.0	8	16.0	20	40.0	30	60.0	20.896	<0.001**
	Follow up	40	80.0	10	20.0	19	38.0	31	62.0	17.199	<0.001**
Hygienic measures	Pre	8	16.0	42	84.0	10	20.0	40	80.0	3.356	0.266
	Post	45	90.0	5	10.0	22	44.0	28	56.0	21.147	<0.001**
	Follow up	40	80.0	10	20.0	20	40.0	30	60.0	15.131	<0.001**
Total self-care domains	Pre	10	20.0	40	80.0	10	20.0	40	80.0	0.000	1.000
	Post	45	90.0	5	10.0	17	34.0	33	66.0	21.616	<0.001**
	Follow up	45	90.0	5	10.0	16	32.0	34	68.0	18.403	<0.001**

### Relationship between age, BMI, parity, delivery methods, and symptoms

Age, BMI, parity, and delivery method had a significant relationship with urinary incontinence symptoms in the study group (Table 6). Obese women were found to have a higher incidence of symptoms during pre-, post, and follow-up interventions. Furthermore, parity and delivery method were also significant predictors of symptoms during the post and follow-up interventions, with women who delivered via normal vaginal delivery and cesarean section having the highest mean of symptoms (Table 6).

**Table 6:** Relationship between follow-up symptoms, sociodemographic characteristics, and previous obstetric history before and after program implementation (study group n=50)

General Characteristics Items	Symptoms of urinary incontinence											
	Pre		F-value	p-value	Post		F-value	p-value	Follow Up		F-value	p-value
	Mean	±SD			Mean	±SD			Mean	±SD		
**Age (years)**												
18-24.9	24.00	0.00	1.529	0.201	43.00	2.31	4.378	0.003*	41.50	2.23	3.984	0.004*
25-34.9	25.93	5.03			40.86	4.59			39.43	4.43		
≥35-60	21.50	1.57			39.17	5.47			37.80	5.28		
**Body Mass Index**												
Normal weight (18.5–25)	20.83	1.40	4.479	0.008*	35.67	4.85	6.203	<0.001**	34.42	4.68	5.645	<0.001**
Overweight (25–30)	21.50	0.53			38.50	5.93			37.15	5.72		
Obesity ( >30)	23.93	3.47			43.14	1.70			41.63	1.64		
**Gravidity**												
1	23.23	3.76	0.364	0.697	39.95	5.03	4.079	0.023*	38.55	4.85	3.712	0.033*
2	22.33	1.37			41.00	4.73			39.57	4.56		
Three or more times	22.00	0.00			33.00	0.00			31.85	0.00		
**Parity**												
1	22.86	2.61	0.20	0.654	37.95	4.88	20.01	<0.001**	39.33	5.06	18.21	<0.001**
2	25.33	7.61			41.81	0.50			43.33	0.52		
Three or more times	22.00	0.00			36.19	5.99			37.50	6.21		
**Methods of delivery**												
Normal vaginal delivery	82.50	0.58	3.352	0.012*	96.00	1.15	8.186	<0.001**	92.64	1.11	7.449	<0.001**
Cesarean section CS	84.86	8.40			94.57	4.40			91.26	4.25		
Instrumental delivery	75.17	4.88			89.67	8.22			86.53	7.93		
Forceps delivery	76.75	2.43			91.75	1.39			88.54	1.34		
Ventouse delivery	71.86	4.95			87.43	9.34			84.37	9.01		

## DISCUSSION

The present study examined the impact of a self-care program on the level of self-care among women with urinary incontinence (UI) in Egypt. In terms of demographic characteristics, most participants in both the study and control groups were married housewives in their late twenties to early thirties, with a higher prevalence of obesity. These findings align with previous studies conducted in Egypt [18], which reported similar demographic profiles among women with UI. However, the study contradicts the findings of Eldeen [19], who reported a higher mean age and lower education level among women with UI. In terms of occupation, the present study found that less than three-quarters of the participants were housewives. This finding suggests that women with urinary incontinence (UI) may not perceive the need for medical advice due to their occupation. However, this finding contradicts a study by Mohammed *et al*. [20] in Minia, Egypt, which reported that more than half of the women studied were employed.

In terms of pregnancy and childbirth characteristics, the study revealed that both groups had a high number of pregnancies, with a higher prevalence of cesarean sections in the study group. This aligns with previous research by Ajith *et al*. [21], which established that pregnancy and childbirth are significant risk factors for urinary incontinence. The impact of pelvic floor muscle damage during vaginal deliveries on the development of various forms of incontinence, such as stress and urge incontinence, cannot be underestimated.

There were no significant differences (p>0.05) between the study and control groups regarding the items related to obstetrics and gynecology history. This finding aligns with a study conducted by Balambika and Sathyaprabha [22] in India, which reported similar results.

When examining the symptoms of urinary incontinence, the study found no significant differences between the study and control groups in all items related to symptoms and types of urinary incontinence. These findings are consistent with studies conducted by Asklund *et al*. [4] in Sweden and Mohamed *et al*. [23] in Egypt, which also reported statistically insignificant differences in symptoms between the study and control groups at the beginning of the intervention.

Regarding the voiding diary, the results of the present study indicated a high level of statistical significance (p<0.001) between the study group and the control group in all items of the voiding diary post-intervention. However, there was a statistical difference (p>0.05) between the study group and control group in all items of voiding diary during the follow-up period. Specifically, the study by Özden *et al*. [24] supports these findings regarding the positive effects of training guidelines on urinary frequency and incontinence.

Regarding women's satisfaction with self-care practices for urinary incontinence before and after implementing the program, the present study demonstrated high statistical significance (p<0.001) between the study group and the control group in all items of the post-program and follow-up domains of self-care practices. However, there was a statistically insignificant association (p>0.05) between the study and control groups in all items of the pre-program domain of self-care practices. These findings are consistent with a study published by Gouda *et al*. [25], which also reported significant improvements in self-care practices among women with UI following a self-care program.

The study found a significant positive correlation between the study group and the control group in terms of total practice scores post-program and during follow-up, indicating the effectiveness of the program in improving self-care levels. These findings are in line with previous studies, such as the study by Helmy *et al*. [26] on the effect of a video-assisted teaching program on pelvic floor muscle exercises in women with urinary incontinence. The positive effect of the program on self-care practices can be attributed to the delivery of information, education, and training on UI management, as well as the promotion of healthy behaviors and habits.

However, the present study contradicts the findings of Sayed *et al*. [27], who reported a high positive correlation between the total level of self-care practices during the pretest, highlighting the need for more research to better understand the factors affecting self-care practices.

Furthermore, the study found a significant difference between the study group and the control group in all items of bladder retraining post-program and during follow-up. This is in line with the findings of Vaz *et al*. [28], who reported the effectiveness of bladder training in improving UI symptoms.

The research also demonstrated the effectiveness of providing adequate information and physical skills training in enhancing confidence and ensuring correct performance of pelvic floor muscle exercises, which is consistent with the findings of Hamzaee *et al*. [29] on the efficacy of the health belief model in improving Kegel exercise practice and self-efficacy in middle-aged women. However, the present study contradicts the findings of Fritel *et al*. on the effectiveness of supervised prenatal pelvic floor exercises in preventing urinary incontinence [30].

Regarding personal hygiene practices, this study found a significant difference between the study group and the control group in all items of practicing personal hygienic measures post-program and during follow-up, consistent with the findings of Asklund *et al*. [4] on the effectiveness of telehealth interventions in improving obstetric and gynecologic health outcomes.

## CONCLUSION

Both the ellipsoid and perimetric methods showed strong and positive correlations for pituitary adenoma volume measurements. However, the ellipsoid method tended to overestimate tumor volume. We found no correlation between adenoma size reduction and the degree of biochemical response in functioning adenomas.
